# COPING STRATEGIES AS MODERATORS OF NEGATIVE HEALTH IMPACTS FROM RELOCATION AT VERY OLD AGE

**DOI:** 10.1093/geroni/igz038.2883

**Published:** 2019-11-08

**Authors:** Bjorn Slaug, Oliver K Schilling

**Affiliations:** 1 Department of Health Sciences, Faculty of Medicine, Lund University, Lund, Sweden; 2 Department of Psychological Ageing Research, Institute of Psychology, Heidelberg University, Heidelberg, Germany

## Abstract

Relocation at very old age is a major life event that may have profound psychological impact. The loss of a home environment that has shaped important aspects of the life course can have negative health impacts, such as lower life satisfaction, depressive symptoms, loss of perceived autonomy and functional independence. However, from an ecological theory perspective, implying that patterns of health and well-being are impacted by a dynamic interplay of personal and environmental factors unfolding throughout the life course, the individual’s adaptive repertoire and resources influence how the individual manages and copes with such major life events as relocation to a new housing environment. To study if coping strategies moderated negative health impacts from relocation at very old age, we utilized longitudinal data of older community-living people from Sweden and Germany who had relocated at some point over a nine-year period (N=79, aged 80+ at baseline). A mixed model approach, adjusting for age at time of relocation, was used to analyze moderating effects of different coping strategies, defined according to Staudinger, Freund & Smith (1995). We found pro-active coping strategies such as reminding oneself of previous ability to solve problems, to significantly moderate negative effects on perceived functional independence and resilient strategies such as letting things just have their course, to significantly moderate negative effects on life satisfaction. We found no significant moderation of depressive symptoms. These results suggest that individual disposition to use different coping strategies can moderate the impact on health that relocation at very old age has.

